# Mitochondrial D-Loop Region Methylation and Copy Number in Peripheral Blood DNA of Parkinson’s Disease Patients

**DOI:** 10.3390/genes12050720

**Published:** 2021-05-12

**Authors:** Andrea Stoccoro, Adam R. Smith, Filippo Baldacci, Claudia Del Gamba, Annalisa Lo Gerfo, Roberto Ceravolo, Katie Lunnon, Lucia Migliore, Fabio Coppedè

**Affiliations:** 1 Department of Translational Research and of New Surgical and Medical Technologies, University of Pisa, 56126 Pisa, Italy; andrea.stoccoro@unipi.it (A.S.); lucia.migliore@unipi.it (L.M.); 2 Institute of Biomedical and Clinical Sciences, University of Exeter Medical School, University of Exeter, Exeter EX2 5DW, UK; A.R.Smith@exeter.ac.uk (A.R.S.); K.Lunnon@exeter.ac.uk (K.L.); 3 Department of Clinical and Experimental Medicine, University of Pisa, 56126 Pisa, Italy; filippo.baldacci@unipi.it (F.B.); claudia.delgamba@gmail.com (C.D.G.); annalisalogerfo2@virgilio.it (A.L.G.); roberto.ceravolo@unipi.it (R.C.); 4 Department of Laboratory Medicine, Azienda Ospedaliero Universitaria Pisana, 56124 Pisa, Italy

**Keywords:** Mitoepigenetics, DNA methylation, D-loop region, Parkinson’s disease

## Abstract

Altered mitochondrial DNA (mtDNA) methylation has been detected in several human pathologies, although little attention has been given to neurodegenerative diseases. Recently, altered methylation levels of the mitochondrial displacement loop (D-loop) region, which regulates mtDNA replication, were observed in peripheral blood cells of Alzheimer’s disease and amyotrophic lateral sclerosis patients. However, nothing is yet known about D-loop region methylation levels in peripheral blood of Parkinson’s disease (PD) patients. In the current study, we investigated D-loop methylation levels and mtDNA copy number in peripheral blood of 30 PD patients and 30 age- and sex-matched control subjects. DNA methylation analyses have been performed by means of methylation-sensitive high-resolution melting (MS-HRM) and pyrosequencing techniques, while mtDNA copy number was analyzed by quantitative PCR. MS-HRM and pyrosequencing analyses provided very similar D-loop methylation levels in PD patients and control subjects, and no differences between the two groups have been observed. Treatment with L-dopa and duration of the disease had no effect on D-loop methylation levels in PD patients. Additionally, mtDNA copy number did not differ between PD patients and control subjects. Current results suggest that D-loop methylation levels are not altered in peripheral blood of PD patients nor influenced by dopaminergic treatment.

## 1. Introduction

Parkinson’s disease (PD) is the second most common neurodegenerative disorder after Alzheimer’s disease (AD), and it is estimated that it currently affects 2–3% of the population over 65 years of age [1]. The two main features of PD include neuronal loss in specific areas of the substantia nigra and the widespread intracellular α-synuclein protein accumulation known as Lewy bodies; the dopaminergic depletion in the nigro-striatal pathway underlies the motor symptoms that characterize patients with PD, including bradykinesia, tremor, rigidity, balance and gait impairment [2,3]. The course of PD is currently unpredictable, and treatment addresses symptoms but does not alter disease progression. Systemic administration of the dopamine precursor L-3,4-dihydroxyphenylalanine (L-dopa) is the gold standard for PD treatment, and, over time, almost all PD patients require this pharmacological treatment [4]. Several pathophysiological mechanisms have been suggested to underlie the neurodegeneration of PD, including mitochondrial impairment, which seems to be an early event in PD [5].

From a genetic perspective, PD can be divided into familial and sporadic forms. Familial PD accounts for only about 5–10% of all cases and results from mutations in one of several causative genes, among which the most frequent are *SNCA*, *LRRK2*, *VPS35*, *PARKIN*, *PINK1,* and *DJ1* [6]. However, for the majority of PD cases, the etiology is multifactorial, involving both genetic and environmental factors, such as pesticides and others [7].

Epigenetic mechanisms, which could be dysregulated by environmental factors, are increasingly discovered to be involved in the pathogenesis and progression of PD [8]. Indeed, several studies revealed global and gene-specific epigenetic changes in the nuclear genome from brain regions of PD animal models and in post-mortem brain regions and peripheral blood from PD patients [9]. Recent evidence suggests that also epigenetic modifications of the mitochondrial genome could contribute to neurodegeneration [10]. In this regard, dysregulation of mitochondrial DNA (mtDNA) methylation in the central nervous system and in peripheral blood samples of patients with AD, PD, and amyotrophic lateral sclerosis (ALS), as well as in transgenic mice modelling, has been reported [11,12,13,14,15,16]. The majority of these studies investigated the methylation levels of the mtDNA regulatory region (D-loop), which regulates mtDNA replication and transcription, revealing that D-loop methylation levels were dynamically dysregulated with disease progression in animal models of AD and were significantly different in human postmortem AD brains and in peripheral blood cells of living AD patients compared to healthy controls [12,13,15]. Altered D-loop methylation levels were also detected in spinal cord and skeletal muscle cells of human-*SOD1* transgenic ALS mice [11] as well as in peripheral blood of familial and sporadic ALS patients [14,16]. Moreover, an inverse correlation between D-loop methylation levels and mtDNA copy number was frequently observed [14,16,17,18,19], suggesting a role for methylation levels in this region in regulating mtDNA replication. Regarding PD, a significant reduction of D-loop methylation levels was observed post-mortem in the substantia nigra of individuals with PD compared to healthy controls [12]. More recently, a study performed in platelet mtDNA did not find differences in the methylation levels of *MT-TL1*, *MT-CO1*, *MT-CO2,* and *MT-CO3* mitochondrial genes between PD patients and control subjects [20]. However, nothing is yet known about D-loop region methylation levels in peripheral blood of PD patients.

In the current study, to further address the potential involvement of mtDNA methylation in PD pathogenesis, we quantified DNA methylation levels in the D-loop regulatory region and mtDNA copy number in peripheral blood cells from sporadic PD patients and matched healthy controls.

## 2. Materials and Methods

### 2.1. Study Population

In the current study 60 individuals, including 30 PD patients and 30 healthy age- and sex-matched controls (Table 1), have been enrolled. All PD patients were recruited at the Neurology Unit of the Clinical and Experimental Medicine Department of the University of Pisa and fulfilled the diagnostic criteria for idiopathic PD [21]. Atypical signs, possibly suggesting atypical parkinsonisms, were considered as exclusion criteria. Information about disease duration and therapy were collected. As normal controls, healthy volunteer subjects matched for age, sex, and ethnic background but with no relationship to the PD patients, have been recruited. Family history of PD was ascertained, excluding all subjects with even one relative who developed PD or any other neurodegenerative disorder. Informed and written consent was obtained from each subject before inclusion in the study that was approved by the Ethics Committee of the Pisa University Hospital (Protocol number 14767/2018). The study was performed in accordance with the Declaration of Helsinki.

### 2.2. D-Loop Methylation Analysis

Genomic DNA was extracted from peripheral blood collected in EDTA tubes from each subject using the QIAmp DNA blood Mini Kit (Qiagen, Milan, Italy, Catalog N° 51106) following the manufacturer’s instructions, and quantified using a Nano Drop ND 2000c spectrophotometer (NanoDrop Thermo scientific, Wilmington, DE, USA). Methylation of the D-loop region was assessed by means of methylation sensitive-high resolution melting (MS-HRM) and pyrosequencing techniques, as reported elsewhere [13,16,22].

A D-loop region of 222 bp, comprising the nucleotides 35–256 of the mtDNA Light (L)-strand (GenBank: J01415.2), which included ten CpG sites, was analyzed by means of MS-HRM, using the primers reported in Table 2. The pyrosequencing analysis was applied to quantify the DNA methylation across three individual CpG sites in the Heavy (H)-strand of the D-loop region, included in a 226 bp amplicon, from the nucleotide 16,417 to the nucleotide 73 within mtDNA (GenBank: J01415.2) by using the primers reported in Table 2. Since there was a high correlation for methylation levels among the three CpGs sites analyzed (r = 0.66, *p* < 0.0001 between CpG1 and CpG2; r = 0.83, *p* < 0.0001 between CpG1 and CpG3; r = 0.85, *p* < 0.0001 between CpG2 and CpG3), the mean methylation levels of the three CpG sites was used for the data analysis.

### 2.3. Assessment of mtDNA Copy Number

The quantification of the mtDNA copy number was performed by means of quantitative PCR (qPCR) by using primers specific for a nuclear DNA region and a mtDNA region [23], as previously reported [14]. To determine the mtDNA content relative to nDNA, the following equations taken from literature [24] were used:

ΔCt = nDNA Ct − mtDNA Ct(1)

Relative mtDNA content = 2 × 2^ΔCt^ (2)

### 2.4. Statistical Analyses

D-loop methylation data and mtDNA copy number were tested for normality using the Kolmogorov–Smirnov test. Demographic data, such as age at sampling and sex, were compared between groups by means of a Student’s t-test and Fisher’s exact test, respectively. Analysis of covariance (ANCOVA), including age at sampling and sex as covariates, was used to investigate differences in D-loop methylation levels and mtDNA copy number among groups. Data are presented as mean ± SEM. Pearson correlation coefficients were used to evaluate correlations between D-loop methylation levels, mtDNA copy number, age at sampling, and disease duration. Statistical analyses were performed with STATGRAPHICS 5.1 plus software package for Windows and the MedCalc statistical software v. 12.5. Figures were obtained with GraphPad PRISM version 6.01. The power of the study was evaluated with the statistical package QUANTO 1.2.4.exe. The present study has an a priori power to detect differences in mean pyrosequencing D-loop methylation levels of about 1.2% between groups. Similarly, the present study has 80% power to detect a mean difference of 40 mtDNA copy numbers between groups.

## 3. Results

### 3.1. D-Loop Methylation Levels and mtDNA Copy Number in PD Patients and Control Subjects

**Figure 1:**
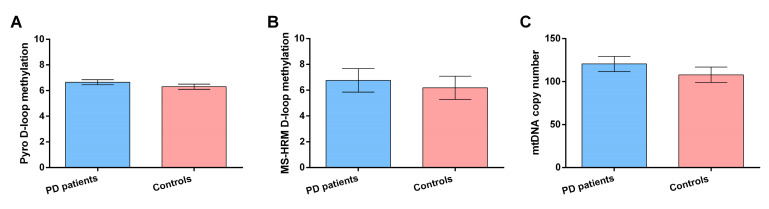
Pyrosequencing (**A**) and MS-HRM (**B**) D-loop region methylation levels and mtDNA copy number (**C**) in PD patients (*n* = 30) and control subjects (*n* = 30). Data are expressed as mean ± SEM. Statistical analysis was performed by means of Analysis of covariance (ANCOVA), including age at sampling and sex as covariates.

In Figure 1A,B are reported results on DNA methylation differences, evaluated by both MS-HRM and pyrosequencing, respectively, between PD patients and control subjects. Analysis of covariance showed that there was no difference in D-loop methylation levels evaluated by means of pyrosequencing between PD patients and control subjects (6.65 ± 0.20% vs. 6.30 ± 0.20%, *p* = 0.29). Similarly, no difference in MS-HRM D-loop methylation levels was observed between PD patients and controls (6.76 ± 0.91 vs. 6.19 ± 0.90%, *p* = 0.66). Interestingly, D-loop methylation levels evaluated by both techniques were very similar, although with MS-HRM, we evaluated the average methylation levels of a fragment of 222 bp containing 10 CpG sites, while with pyrosequencing, we focused on three CpG sites within this region. In Figure 1C are reported results on mtDNA copy number mean methylation difference between control subjects and PD patients. Analysis of covariance showed that there was not a statistically significant difference in mtDNA copy number between PD patients and control subjects (120.51 ± 8.96 vs. 107.84 ± 8.09, *p* = 0.33). Sex had no effect on either D-loop methylation levels or mtDNA copy number in our population, while age showed an inverse correlation with pyrosequencing D-loop methylation levels (r = −0.52, *p* < 0.0001), and a weak inverse correlation was observed between MS-HRM D-loop methylation and mtDNA copy number (r = −0.29; *p* = 0.02).

### 3.2. Effect of Levodopa Use and Disease Duration on DNA Methylation Levels

**Figure 2:**
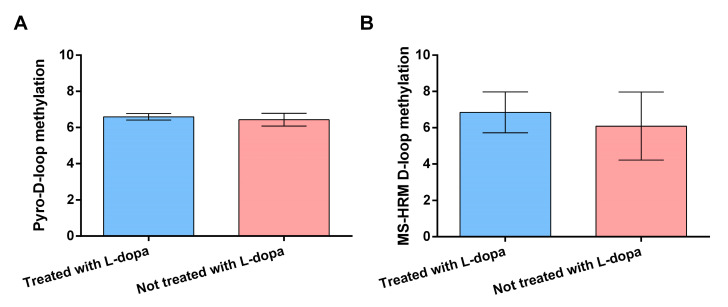
Pyrosequencing (**A**) and MS-HRM (**B**) D-loop region methylation levels in PD patients treated with L-dopa (*n* = 22) and PD patients not treated with L-dopa (*n* = 8). Data are expressed as means ± SEM. Statistical analysis was performed by means of ANCOVA, including age at sampling and sex as covariates.

In Figure 2A,B are reported results on the effects of use of L-dopa on D-loop methylation levels evaluated by means of pyrosequencing and MS-HRM, respectively. No significant difference in pyrosequencing D-loop methylation levels was observed between PD patients treated or not treated with L-dopa (6.43 ± 0.35 vs. 6.59 ± 0.18, *p* = 0.71). Similarly, no significant difference was observed in MS-HRM D-loop methylation levels between the two aforementioned groups (6.09 ± 1.88 vs. 6.85 ± 1.20, *p* = 0.73).

**Figure 3:**
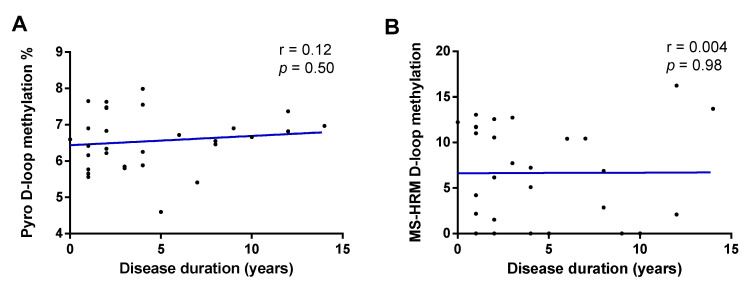
Correlation between disease duration (in years) and D-loop region methylation levels evaluated by means of pyrosequencing (**A**) and MS-HRM (**B**). The correlation was analyzed using Pearson’s correlation coefficient.

In Figure 3A,B are reported results on the effect of disease duration (in years) on D-loop methylation levels evaluated by means of pyrosequencing and MS-HRM, respectively. No significant effect of disease duration was observed on D-loop methylation levels evaluated by means of pyrosequencing (r = 0.12; *p* = 0.50) and MS-HRM (r = 0.004; *p* = 0.98).

## 4. Discussion

Owing to the central role of mitochondria in regulating cell metabolism, the investigation of mitochondrial epigenetics is a recent and attractive field in human diseases and many authors have searched for mtDNA methylation differences in blood and brain samples from patients and animal models of neurodegeneration compared to healthy controls [10]. Particularly, the D-loop region regulates transcription and replication of the mtDNA, and several investigators observed an inverse correlation between D-loop methylation levels and the mtDNA copy number [14,16,17,18,19]. Indeed, changes in D-loop methylation levels, often paralleled by changes in mtDNA copy number, have been observed in AD blood samples [13], post-mortem AD and PD brains [12], AD animal models [12], ALS mice [11], and blood samples from patients with both familial *SOD1* ALS and sporadic ALS [14,16]. Conflicting results have so far been obtained when searching for mtDNA copy number differences in blood samples of PD patients compared to matched controls [20,25,26,27,28], but no previous study has evaluated D-loop methylation levels in peripheral blood DNA samples of PD patients. In the current study, D-loop methylation levels evaluated by means of pyrosequencing and MS-HRM techniques and the mtDNA copy number have been investigated in peripheral blood of PD patients and of neurologically healthy controls. We did not observe differences in D-loop methylation levels and in mtDNA copy number between PD patients and control subjects. A strong inverse correlation between D-loop methylation levels and age at sampling (evaluated by means of pyrosequencing) and a negative correlation between D-loop methylation levels and the mtDNA copy number (evaluated by means of MS-HRM) were detected. Use of L-dopa and disease duration did not affect D-loop methylation levels in PD patients.

Until now, two studies evaluated mtDNA methylation levels in specimens from individuals with PD. Blanch and collaborators investigated the methylation levels of D-loop region and of *MT-ND6* gene in the substantia nigra of 10 PD patients and 10 control subjects, observing decreased methylation levels of D-loop in PD patients [12]. In a more recent study, no differences in methylation levels of the mitochondrial genes *MT-TL1*, *MT-CO1*, *MT-CO2,* and *MT-CO3* in platelet mtDNA from 47 PD patients and 40 control subjects were observed [20]. Although we analyzed a different mitochondrial region, our results are in line with those obtained by Sharma and coworkers, who overall suggested that peripheral mtDNA methylation is not altered in PD patients. Regarding the results obtained by Blanch and coworkers [12], who observed decreased D-loop methylation levels in PD patients, it should be underlined that they analyzed mtDNA extracted from substantia nigra from the PD post-mortem brain, and it is well known that epigenetic mechanisms, including DNA methylation, are tissue-specific so that changes observed in a particular brain region are not always mirrored by similar changes in peripheral tissues [29]. 

We also evaluated the effect of L-dopa and of disease duration on D-loop methylation levels given that L-dopa could affect DNA methylation levels of specific nuclear genes [30,31,32] and that D-loop methylation levels tend to decrease with disease progression in AD animal models [12]. For example, *SNCA* methylation was found to be increased in peripheral blood of PD patients who received higher L-dopa dosage, suggesting that its pharmacological action was not limited to the dopamine precursor function but included epigenetic off-target effects detectable also in peripheral blood [30]. Moreover, chronic L-dopa exposure induced CpG hypermethylation in the *MAO-A* promoter, a gene involved in dopamine metabolism, while acute L-dopa administration resulted in *MAO-A* hypomethylation in neuronal SH-SY5Y cells [31]. In a longitudinal genome-wide methylation study covering ~850,000 nuclear genome CpG sites performed in peripheral blood of 189 PD patients obtained at baseline and at a follow-up visit after two years, 237 CpG sites were identified whose methylation levels changed longitudinally over time [32]. Interestingly, DNA methylation levels of 214 CpG sites associated with disease duration were affected by the dopaminergic medication, which included L-dopa and entacapone, thus emphasizing that DNA methylation is dynamic in PD and that common PD medication could impact methylation of specific CpG sites of nuclear DNA [32]. However, we did not observe different D-loop methylation levels between patients treated or not with L-dopa, and no correlation of D-loop methylation levels with disease duration was identified, thus suggesting that, unlike nuclear DNA methylation, mitochondrial D-loop methylation in peripheral blood cells of PD patients is not sensitive to PD progression and medication.

In the current study, no difference in mtDNA content between PD patients and control subjects was detected. Several authors used mitochondrial copy number as a measure of mitochondria health status in PD tissues, including blood and brain samples. However, results from those studies are largely discordant and no clear conclusions have yet been reached on whether or not mtDNA copy numbers are impaired in PD tissues [20,25,26,27,28,33,34,35,36,37].

D-loop is a non-coding mitochondrial region of about 1.1 kb; it is critical for mitochondrial replication since it contains the origin of replication of the heavy mtDNA strand, making this mitochondrial region a valuable candidate for epigenetic modifications [38]. In this regard, several authors searched for relationships between D-loop methylation levels and mtDNA copy number, reporting an inverse correlation between the two markers in human and mouse cell cultures [38,39], in human peripheral blood cells [14,16,19,40,41], in colorectal cancer tissues [17], and in the human placenta [18,42]. We observed an inverse correlation between MS-HRM D-loop methylation levels and mtDNA copy number, further suggesting that variations in D-loop methylation levels could modulate the mtDNA copy number. In the current study, we also observed that age at sampling inversely correlated with pyrosequencing D-loop methylation levels, an observation that confirmed a previous report by us [16]. In addition, other authors observed that mtDNA methylation evaluated in peripheral blood changed with age. In a study performed in 381 individuals aged from 38 to107 years, the methylation levels of the *MT-RNR1* gene were positively associated with increasing age [43]. Moreover, after a nine-year-long follow-up survey, the authors also showed that subjects with high methylation levels exhibited a mortality risk higher than subjects with lower *MT-RNR1* methylation levels [43]. A subsequent bisulfite sequencing study of mtDNA was performed in peripheral blood of 82 individuals aged 18–91 years, and methylation levels of two CpG sites located within the *MT-RNR1* gene were inversely correlated with age [44]. Overall, these studies suggested that peripheral blood mtDNA methylation could be a useful epigenetic mark of ageing. However, due to the fact that D-loop methylation levels were very similar between PD patients and healthy controls (when quantified using either pyrosequencing or MS-HRM methodologies), they are unlikely to represent peripheral epigenetic biomarkers of PD. On the other hand, epigenetic modifications of D-loop deserve a role in discriminating PD from other neurodegenerative disorders and could be a potential tool to predict the development of PD dementia as well. Indeed, growing evidence charted significant epigenetic alterations of D-loop region in AD compared to controls [12,13,15]. Cross-sectional and prospective studies evaluating potential epigenetic differences in the D-loop region of PD and AD populations are warranted, especially for specific subsets of PD populations where subtle cognitive impairment has been reported since disease onset [45].

The use of two complementary techniques for the investigation of the D-loop methylation levels is the main strength of the current study. MS-HRM is a simple, closed tube, and relatively inexpensive method that has been shown to be highly specific and highly sensitive when a proper PCR protocol is applied [46,47]. However, one of the main disadvantages of MS-HRM is that it does not provide information on methylation levels of single CpGs but only provides the mean methylation level of the amplicon analyzed. On the other hand, pyrosequencing is considered the gold standard technique for candidate gene methylation studies, providing information on the methylation levels of single CpG sites. MS-HRM and pyrosequencing provided very similar results regarding mean D-loop methylation levels in PD patients and in control subjects despite the fact they analyzed a different number of CpG dinucleotides; specifically, 3 CpG sites were covered by the pyrosequencing, while 10 CpG sites were covered by the MS-HRM, thus reinforcing the evidence that D-loop methylation levels are not altered in the peripheral blood of PD patients. 

In summary, we performed the present study searching for mtDNA methylation and copy number differences between PD and control blood DNA samples. The present investigation suggests that D-loop methylation levels are not altered in blood DNA samples from PD; thus, it unlikely represents a peripheral methylation biomarker of PD. One of the limitations of the present study is the relatively small sample size of the case-control cohort. However, with a case-control cohort of 30 samples, each the study has enough power to detect differences in mean pyrosequencing D-loop methylation levels of about 1.2% between groups. Similarly, the study has enough power to detect a mean difference of about 40 mtDNA copy numbers between groups. Average D-loop methylation differences of about 1% or higher have been previously observed in the comparison of patients with neurodegenerative diseases and healthy matched controls [14,16], and the present study has enough power to detect those differences. Furthermore, in the present cohort, there was also no trend towards decreased D-loop methylation levels in PD patients compared with controls. However, we have estimated that cohorts composed by at least 45 cases and 45 controls are required to detect smaller differences in D-loop methylation levels (<1% in average) between groups. Therefore, further studies empowered in sample size are required prior to exclude that slight differences in D-loop methylation levels might account for a small variability in the mtDNA copy number between PD patients and controls. Moreover, we cannot exclude that other mtDNA regions could be affected by aberrant methylation in PD peripheral samples, although in another report, no altered methylation of four mtDNA genes, namely *MT-TL1*, *MT-CO1*, *MT-CO2,* and *MT-CO3*, were observed in the platelets of PD patients [20]. Epigenetic changes are tissue-specific and studies in PD-affected brain tissues are required prior to exclude a contribution of impairment of mtDNA methylation in PD pathogenesis. In line with this consideration, a pilot study performed in the substantia nigra of ten PD post-mortem brains identified decreased D-loop methylation levels when compared to age and sex-matched control subjects’ brain tissues [12]; accordingly, mitochondrial epigenetic alterations deserve to be further investigated in PD. Moreover, we did not observe alteration in mtDNA copy number in PD peripheral blood cells. However, considering the conflicting nature of previous literature investigations, further studies also including neurodegenerative atypical parkinsonisms are needed to shed light on the potential mtDNA copy number changes and the epigenetic modifications in PD.

## 5. Conclusions

In conclusion, results of the current study showed that there are no differences in D-loop methylation levels and in mtDNA content, evaluated in peripheral blood, between PD patients and control subjects. Moreover, current results demonstrate that treatment with L-dopa and duration of the disease had no effect on D-loop methylation levels in PD patients.

## Figures and Tables

**Table 1:** Demographic characteristics of the study population.

	Control Subjects (*n* = 30)	PD Patients (*n* = 30)	*p*-Value
Age (mean ± SD)	64.2 ± 14.0	67.7 ± 10.4	0.27 ^a^
Gender (F/M)	11/19	9/21	0.58 ^b^
Treatment with Levo-dopa (Yes/No)	−	22/8	−
Age of onset	−	63.4 ± 10.1	−
Duration of disease (in months)	−	52.8 ± 46.5	−

^a^ Student’s t-test; ^b^ Fisher’s exact test. Data are presented as mean ± SD.

**Table 2:** Primers sequences, amplicon size, and number of CpGs analyzed to investigate D-loop methylation by means of MS-HRM and pyrosequencing.

Technique	Primer Forward	Primer Reverse	Sequencing Primer	Amplicon Size	Number of CpGs
MS-HRM	5′GGAGTTTTTTATGTATTTGGTATTTT-3’	5′ACAAACATTCAATTATTATTATTATATCCT-3’		222 bp	10
Pyrosequencing	Bio5′TAGGATGAGGTAGGAATTAAAGATAGATA-3’	5′ACATCTAATTCCTACTTCAAAATCAT-3′	5′CAAATCTATCACCCTATTAA-3′	226 bp	3

## Data Availability

The datasets generated and/or analyzed during the current study are available from the corresponding author on reasonable request.
